# Case Report: Single-port thoracoscopic surgery for severe primary palmar hyperhidrosis in a 9-year-old child

**DOI:** 10.3389/fmed.2025.1542064

**Published:** 2025-04-15

**Authors:** Zhen Luo, Yu Li, Guangxu Zhou, Kaiyi Mao, Hongyang Tan, Peng Zhao, Yingbo Li, Xianhui Shang, Hong Ma, Cao Wang

**Affiliations:** ^1^Department of Pediatric Surgery, Affiliated Hospital of Zunyi Medical University, Zunyi, Guizhou, China; ^2^Guizhou Children's Hospital, Zunyi, Guizhou, China

**Keywords:** primary palmar hyperhidrosis (PPH), endoscopic thoracic sympathectomy (ETS), child, case report, single-port

## Abstract

Primary palmar hyperhidrosis (PPH) is a somatic condition characterized by excessive sweating of the hands. It mainly affects adolescents and young adults and is rarely observed among children. This condition significantly impairs patients' academic performance, daily activities, and social interactions and can even lead to insurmountable psychological burdens. Surgical intervention for PPH is typically reserved for individuals aged 16 years and older, as compensatory hyperhidrosis occurs at a high rate (65%) postoperatively among children younger than 14 years. Therefore, the decision for surgery is controversial and has been rarely documented in the literature. Here, we report a 9-year-old child with a 3-year history of bilateral palmar hyperhidrosis. Conservative treatment with medications for 6 months led to no improvement. The patient had signs of low self-esteem, social withdrawal, and aversion to school, alongside recurrent skin damage at the fingertips. Both the child and parents expressed a strong desire for effective treatment. Single-port endoscopic thoracic sympathectomy (ETS) was conducted after comprehensive risk disclosure and obtaining informed consent from the parents, achieving remarkable therapeutic outcomes. At the 12-month follow-up, the patient exhibited no recurrence of symptoms, no compensatory hyperhidrosis, and no complications, such as Horner's syndrome. Both hands remained warm and dry, the lesions of fingertip skin healed, and the patient's personality became noticeably more positive. Furthermore, the surgical incision was aesthetically pleasing.

## Highlights

- Primary severe palmar hyperhidrosis can significantly affect the physical and mental health of children, and conservative treatment often yields poor results.
- Primary severe palmar hyperhidrosis in children can be treated surgically.
- Single-port thoracoscopic surgery for primary severe palmar hyperhidrosis in children is safe and effective.

## Background

Palmar hyperhidrosis is the most common subtype of hyperhidrosis, characterized by sweat gland secretion exceeding physiological requirements. It can be classified as either secondary or primary types. Secondary hyperhidrosis often occurs subsequent to conditions such as thyroid disorders, endocrine imbalances, and pituitary tumors, whereas the etiology of primary palmar hyperhidrosis (PPH) remains unknown (1–3). PPH can be further categorized into mild, moderate, and severe PPH. Mild PPH manifests as palm moistness, moderate PPH manifests as small beads on the palms subsequent to sweating (4), and severe PPH manifests as sweat dripping from the palms. A study from Israel indicates that the incidence of hyperhidrosis worldwide is 0.6–1.0% (5).

PPH can be a debilitating condition among children, with an unknown etiology. There exist various treatment options, including topical therapies, systemic medications, iontophoresis, botulinum toxin type A injections, and surgical interventions (6, 7). These approaches often show limited efficacy, have intolerable side effects, and result in poor adherence in pediatric patients, failing to provide a definitive cure. Surgery may offer the best option; however, the optimal time for surgery is typically between the ages of 16 and 50. Symptoms may also evolve in children younger than 16, and compensatory hyperhidrosis is highly likely (65%) after surgery in this age group. Therefore, an observation period is recommended, and no expert consensus has been established (8).

In this case, a 9-year-old boy with severe PPH experienced significant impairments in social interaction, daily life, academic performance, and psychological wellbeing. Persistent cold hands led to recurrent breakdown of fingertip skin. With strong parental support for surgery and after thorough multidisciplinary discussion, endoscopic thoracic sympathectomy (ETS) was conducted using a single-port thoracoscopic approach.

## Case details

A 9-year-old boy presented with a 3-year history of excessive, cold sweating in both hands and was diagnosed with PPH. Medical and physical therapies yielded no improvement. Symptoms had worsened over the last 6 months before presentation, with sweat dripping from his hands, cold and moist fingertips, and associated personality changes, including social withdrawal, irritability, low self-esteem, aversion to school, and refusal to interact with peers.

**Figure 1:**
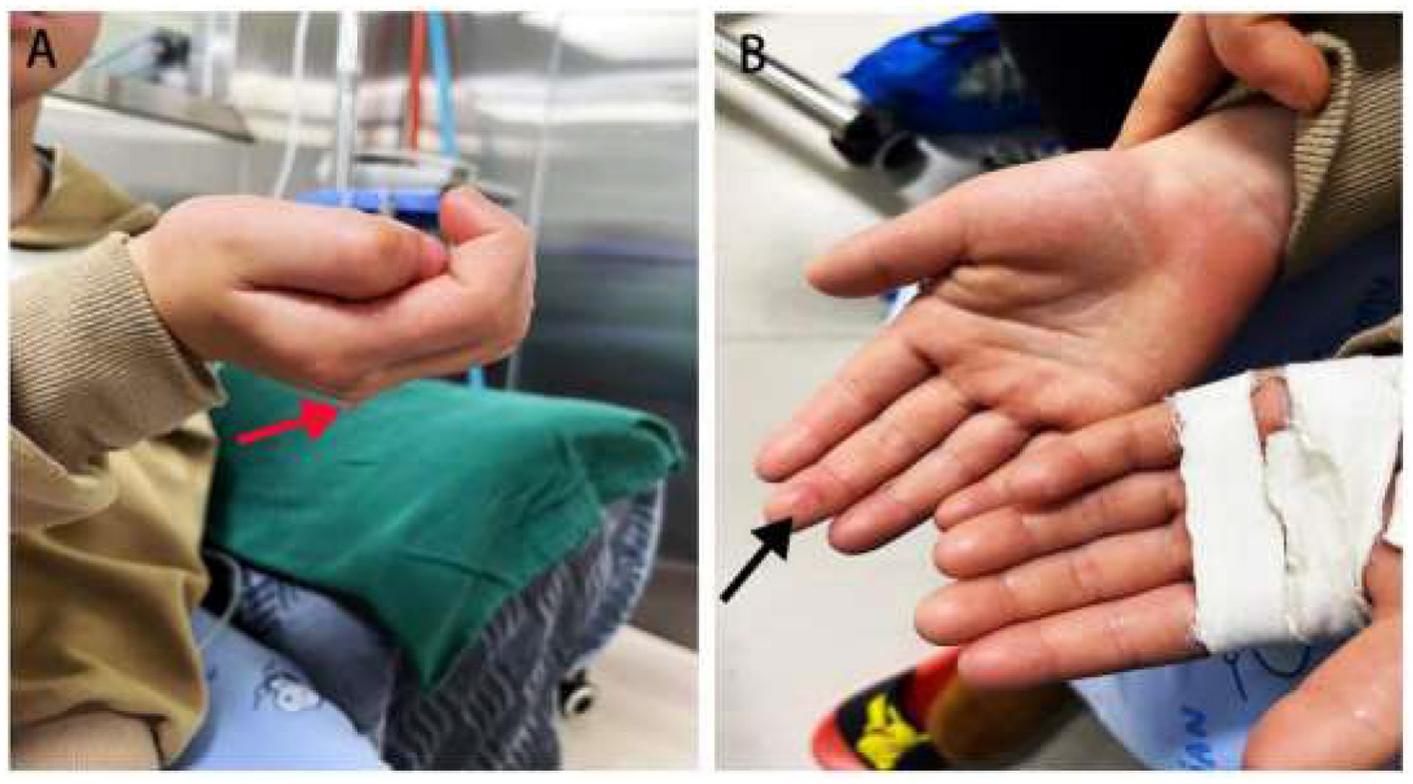
Clinical manifestations of both hands before surgery. **(A)** Profuse sweat dripping from both hands in a natural state (red arrow). **(B)** Skin breakdown on the right middle fingertip with purple discoloration (black arrow).

On physical examination, the child appeared indifferent and hesitant to engage in conversation. Both hands exhibited profuse sweat dripping (Figure 1A), were cold to the touch, and exhibited fingertip skin damage, including purple discoloration and skin breakdown on the right middle finger pad (Figure 1B). There was no increased sweating on the forehead, axillae, chest, back, or groin regions.

### Auxiliary tests

Chest computed tomography (CT) showed no sign of mediastinal tumors. Cranial CT revealed no pituitary abnormalities. Thyroid function was normal based on serum testing. Secondary palmar hyperhidrosis was ruled out. The diagnosis of severe primary palmar hyperhidrosis was confirmed. Conservative treatment with systemic medications was ineffective, and the patient developed compromised fingertip blood circulation and personality changes. With informed parental consent, single-port thoracoscopic sympathetic chain transection was performed.

### Surgical procedure

The patient was placed in a supine position with elevated spine support. Double-lumen endotracheal intubation was employed under general anesthesia. Both arms were abducted. After sterilization and sterile draping of the surgical area, the operating table was adjusted to a high-right, low-left, head-up, foot-down position. The right side was addressed first, followed by the left.

### Right-side approach

**Figure 2:**
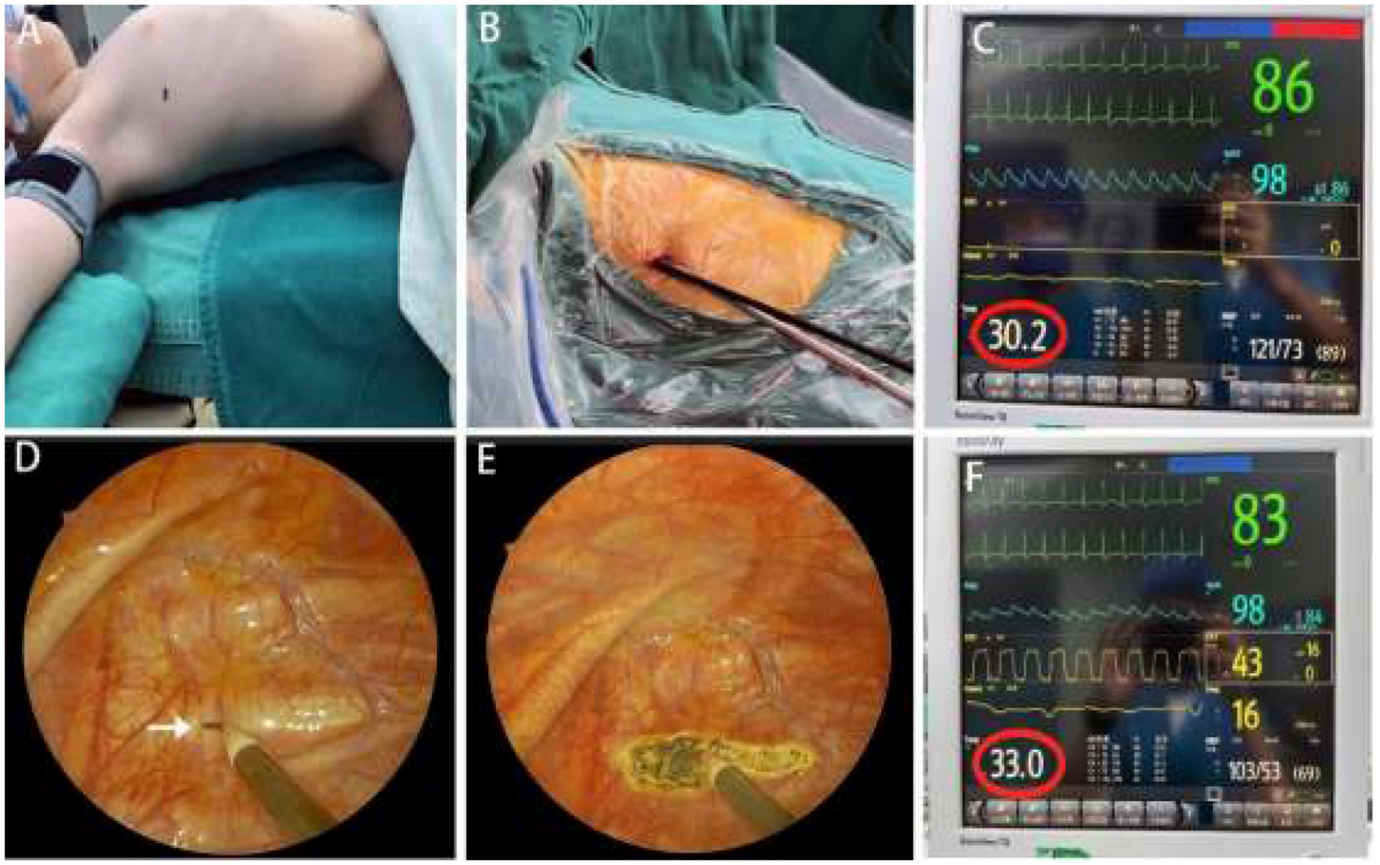
Intraoperative details. **(A)** Patient positioning for surgery, with the black dot indicating the incision site. **(B)** Thoracoscope and electrocautery hook at the fourth intercostal space along the midaxillary line. **(C)** Palm temperature before sympathectomy: 30.2°C (red circle). **(D)** R3 plane identification at the second intercostal space (white arrow). **(E)** R3 sympathetic nerve transection with an electrocautery hook, with the white arrow pointing to the R3 sympathetic nerve. **(F)** Palm temperature after sympathectomy: 33.0°C (red circle).

A 0.5 cm incision was made at the fourth intercostal space along the mid-axillary line (Figure 2A), followed by layer-by-layer dissection into the thoracic cavity (Figure 2A). A 3 mm thoracoscope and a 3 mm electrocautery hook were simultaneously inserted through the incision (Figure 2B). The sympathetic chain at the third rib head was identified after lung deflation. The sympathetic nerve was transected, and electrocautery ablation was extended 5–6 cm along the third rib to cut nerve branches (Figures 2D, E).

During surgery, the pre-sympathectomy palm temperature was 30.2°C (Figure 2C). After transecting the T3 nerve, the palm temperature increased to 33.0°C (Figure 2F). No bleeding or lung injury was observed. A 10-Fr rubber tube was placed in the thoracic cavity. The anesthesiologist was instructed to ventilate and inflate the right lung for re-expansion. The rubber tube's end was placed in water to ensure complete air evacuation, after which it was promptly withdrawn. The incision was closed without placing a chest tube.

The left side was treated following an identical procedure.

**Figure 3:**
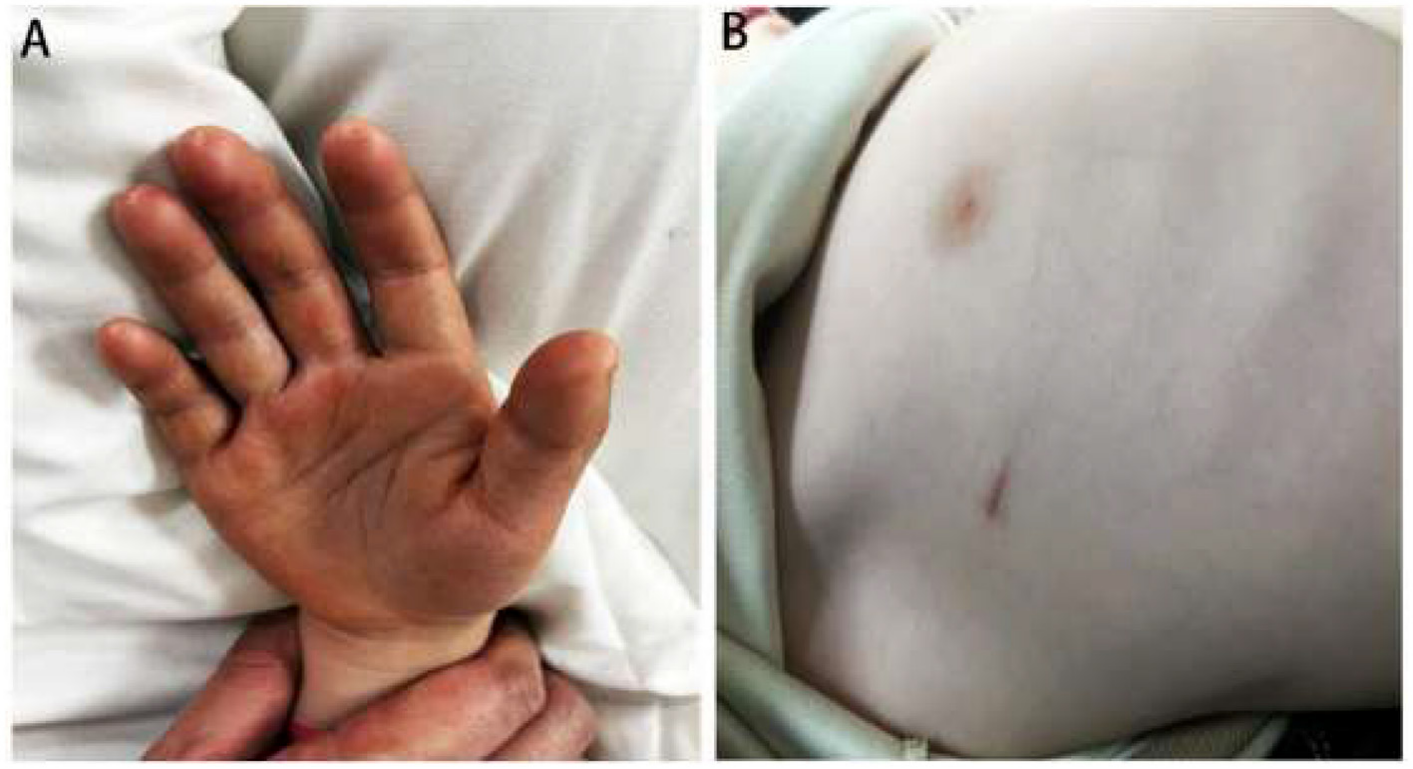
Postoperative outcomes of the pediatric patient. **(A)** Dry, warm palms immediately after surgery. **(B)** The appearance of the surgical incision 1 month after the surgery from the aesthetic perspective.

The surgery was completed successfully in 30 min, with an estimated blood loss volume of 2 mL. On the same day, sweating ceased, and both hands became dry, warm, and reddish (Figure 3A). The patient was discharged 1 day after surgery when chest X-ray excluded pneumothorax. At 1 week, 3 months, 6 months, and 12 months postoperatively, the patient exhibited no recurrence of sweating, compensatory hyperhidrosis, or complications. Fingertip skin lesions healed, and the child's self-esteem, confidence, and social interaction improved (Table 1). Furthermore, the surgical incision remained aesthetically pleasing (Figure 3B).

**Table 1:** Summary of case data.

**General information**	**Result**
Gender	Male
Age	9 years old
Diagnosis	Primary palmar hyperhidrosis
Surgical approach	Single-port thoracoscopic T3 sympathectomy
Preoperative palm temperature	30.2°C
Intraoperative palm temperature	33°C
Palm dryness	Dry
Presence of compensatory hyperhidrosis 1 year after surgery	None
Incision satisfaction	Satisfied
Postoperative personality change	Outgoing personality

## Discussion

This report presents the treatment and outcomes of a 9-year-old child with severe PPH who underwent single-port ETS. The novelty of this case lies in the patient's young age and the severity of symptoms, which significantly affected his daily life and psychological wellbeing. Hyperhidrosis, a common type of localized excessive sweating, is prevalent among children and adolescents. Although its exact etiology remains unclear, it is believed to be associated with the overactivity of the sympathetic nervous system.

Previous studies have explored various treatment options for hyperhidrosis, including topical medications, iontophoresis, botulinum toxin injection, and surgical interventions (9). However, surgical treatment in younger patients, particularly among those as young as 9 years, has been rarely reported. Most studies recommend surgery for patients aged 16 years and older, emphasizing caution among children younger than 16 years due to ongoing physical and psychological development, high surgical risks, and difficulties in adapting to postoperative compensatory hyperhidrosis. Exceptions are typically reserved for patients with severe complications or significant impairment of physical and mental development. Our patient's severe symptoms substantially disrupted social interaction, academic performance, and psychological health. Persistent low palm temperatures led to recurrent fingertip skin damage, highlighting the necessity of early intervention.

Various surgical techniques are available for PPH, with approaches including single-port and multi-port access. The surgical modalities include thoracoscopic sympathectomy, sympathetic nerve clipping, nerve disconnection, and staged procedures (10). Currently, single-session bilateral thoracoscopic T3 sympathectomy is considered an effective treatment option for severe PPH (9). Ongoing research on the pathogenesis and mechanisms of primary palmar hyperhidrosis (PPH) drives innovation in non-surgical treatments like topical medications, iontophoresis, and botulinum toxin injections. Conservative treatments, though less definitive, require repeated use, and have side effects, offer a non-invasive and convenient choice for mild-symptom patients or those unfit for surgery. Surgical intervention, invasive and costly with a risk of compensatory hyperhidrosis, provides a definitive cure with rapid relief and is preferred by patients with moderate- to -severe PPH. Our patient supports this approach, showcasing the feasibility and safety of single-port thoracoscopic surgery for pediatric patients, a method rarely reported in this population. Compared to traditional multi-port procedures, single-port surgery is less invasive and leads to faster recovery and less postoperative pain.

Different levels of sympathetic nerve transection are needed for various forms of focal hyperhidrosis. T2 transection is preferred for severe craniofacial hyperhidrosis, and T3 transection is recommended for moderate craniofacial hyperhidrosis. T3 or T4 transection is recommended for severe palmar hyperhidrosis, while moderate palmar hyperhidrosis generally necessitates T4 transection. Electrocautery is the preferred method for nerve disconnection. However, T2 transection is avoided except for severe craniofacial hyperhidrosis (11). It is necessary to ensure sufficient cauterization of the rib surface to prevent recurrence due to the presence of Kuntz fibers or accessory branches (12).

For this patient, we performed a single-session bilateral T3 sympathectomy with a cauterization width of ~5–6 cm. The intraoperative increase in palm temperature was used as a key indicator of successful nerve transection, and an increase of ~3°C was considered satisfactory (13). For this case, palm temperature rose from 30.2 to 33°C during surgery, indicating effective nerve disruption and achieving favorable outcomes.

Indications for ETS mainly include severe hyperhidrosis unresponsive to conservative treatment, especially severe hyperhidrosis leading to significant psychological or social impairment. In this case, the patient received pharmacological treatment before surgery, which was successful. Given the substantial effect of PPH on daily life and psychological health and the recurrent fingertip skin damage, we employed single-session bilateral T3 sympathectomy using a single-port thoracoscopic approach. After the surgery, the patient exhibited marked improvement, evidenced by dry palms and significantly enhanced quality of life.

It is important to acknowledge the risks associated with ETS. Compensatory hyperhidrosis is the most common postoperative complication, with a reported incidence rate of ~65% among patients younger than 16 years (7). However, we believe that surgical treatment for severe PPH in children under 16 years of age should be approached with caution. Preoperative discussions with both the patient and their family are essential. In this case, no significant compensatory hyperhidrosis was observed during follow-up, although long-term monitoring is essential to assess sustained outcomes and potential complications.

This case highlights the importance of early recognition and intervention for severe PPH among children. In addition to affecting physiological function, PPH severely impairs psychological health and social interactions. For pediatric patients with severe symptoms unresponsive to conservative management, early surgical intervention may be an effective treatment option. The application of single-port thoracoscopic techniques has enhanced surgical safety and patient comfort, offering a more favorable therapeutic solution for pediatric patients.

Despite the positive outcomes in this case, further large-scale clinical studies and long-term follow-up data are necessary to validate the safety and efficacy of single-port thoracoscopic surgery in children. Future research should focus on several key areas: surgical outcomes and postoperative complication rates across different pediatric age groups; comparative studies between single-port thoracoscopic surgery and other surgical techniques; and long-term follow-up data, particularly regarding the incidence and severity of compensatory hyperhidrosis.

## Conclusion

This report details the successful treatment of a 9-year-old child with severe primary palmar hyperhidrosis using single-port thoracoscopic sympathectomy. The case underscores the importance of early intervention and supports the application of single-port thoracoscopic surgery in pediatric patients. We found that single-port thoracoscopic surgery is a safe and effective therapeutic option for children with severe hyperhidrosis who do not respond to conservative treatment.

## Data Availability

The original contributions presented in the study are included in the article/supplementary material, further inquiries can be directed to the corresponding author.
